# Inhibition of dendritic cell migration by transforming growth factor-β1 increases tumor-draining lymph node metastasis

**DOI:** 10.1186/1756-9966-31-3

**Published:** 2012-01-10

**Authors:** Kazuhiro Imai, Yoshihiro Minamiya, Souichi Koyota, Manabu Ito, Hajime Saito, Yusuke Sato, Satoru Motoyama, Toshihiro Sugiyama, Jun-ichi Ogawa

**Affiliations:** 1 Department of Chest, Breast and Endocrinologic Surgery, Akita University Graduate School of Medicine, 1-1-1 Hondo Akita City 010-8543, Japan; 2 Department of Biochemistry, Akita University Graduate School of Medicine, 1-1-1 Hondo Akita City 010-8543, Japan

**Keywords:** dendritic cell, migration, transforming growth factor-β1, tumor draining lymph node, lymph node metastasis

## Abstract

**Background:**

Transforming growth factor (TGF)-β is known to be produced by progressor tumors and to immobilize dendritic cells (DCs) within those tumors. Moreover, although TGF-β1 has been shown to promote tumor progression, there is still no direct, in vivo evidence as to whether TGF-β1 is able to directly induce distant metastasis.

**Methods:**

To address that issue and investigate the mechanism by which TGF-β1 suppresses DC activity, we subdermally inoculated mouse ears with squamous cell carcinoma cells stably expressing TGF-β1 or empty vector (mock).

**Results:**

The numbers of DCs within lymph nodes draining the resultant TGF-β1-expressing tumors was significantly lower than within nodes draining tumors not expressing TGF-β1. We then injected fluorescently labeled bone marrow-derived dendritic cells into the tumors, and subsequent analysis confirmed that the tumors were the source of the DCs within the tumor-draining lymph nodes, and that there were significantly fewer immature DCs within the nodes draining TGF-β1-expressing tumors than within nodes draining tumors not expressing TGF-β1. In addition, 14 days after tumor cell inoculation, lymph node metastasis occurred more frequently in mice inoculated with TGF-β1 transfectants than in those inoculated with the mock transfectants.

**Conclusions:**

These findings provide new evidence that tumor-derived TGF-β1 inhibits migration of DCs from tumors to their draining lymph nodes, and this immunosuppressive effect of TGF-β1 increases the likelihood of metastasis in the affected nodes.

## Background

Transforming growth factor (TGF) -β can reportedly promote cancer metastasis by affecting the tumor microenvironment in a manner that facilitates tumor cell invasion [1,2] and by inhibiting immune cell function [3]. Consistent with those reports, overproduction of TGF-β by tumors is frequently associated with metastasis [4-6] and a poor prognosis in patients with cancer [7-10]. Among the three highly homologous TGF-β isoforms, TGF-β1 is the most abundant and most extensively studied [11]. We previously showed that tumor-derived TGF-β1 causes a reduction in the number of dendritic cells (DCs) within tumor-draining lymph nodes (TDLNs) [12]. It also has been shown that TGF-β1 is produced by progressor tumors and that it immobilizes the DCs within those tumors [13]. This is noteworthy because DCs are highly specialized, antigen-presenting cells that play a crucial role in the initial activation and subsequent regulation of immune responses, and are important for the induction of tumor immunity; they take up antigen within the tumor and migrate to local lymph nodes, where they present the antigen to T cells, inducing immunity [14]. DCs can present antigen in an immunogenic or tolerogenic manner and are a crucial determinant of the host response to tumors. Indeed, tumors are immunologically destroyed when DCs are able to take up antigen and migrate to the lymph nodes, but escape destruction if the DCs are subverted so that they do not migrate to the draining lymph nodes, or if macrophages become the major cell taking up antigen [13,14].

In addition, Cui et al. found that expression of the TGF-β1 transgene inhibited benign tumor formation, but enhanced progression of carcinomas [15]. It is still not known at which stage or by what mechanisms TGF-β1 switches from a tumor suppressor to a tumor promoter. Moreover, no direct in vivo evidence documenting whether TGF-β1 directly induces distant metastasis has yet been reported. To address these issues, we generated a carcinoma stably overexpressing a TGF-β1 transgene. Here we provide in vivo evidence that expression of TGF-β1 may directly induce metastasis in tumors that escape the immune response of DCs, and that down-regulation of DC migration from the tumor to its TDLNs is a key event fostering metastasis.

## Materials and methods

### Mice

Male 6-week-old syngeneic C3H/He N mice were obtained (The Jackson Laboratory, Bar Harbor, Maine) and maintained in accordance with the guidelines of the Committee on Animals of the Akita University School of Medicine.

### Tumor cell lines

SCCVII is a spontaneously arising squamous cell cancer of C3H mice. SCCVII cells were maintained at 37°C in complete medium (CM: RPMI-1640 medium with 2 mM L-glutamine and 1 mM sodium pyruvate) supplemented with 10% FBS, 100 units/ml penicillin G, 0.1 mg/ml streptomycin and 0.5 μg/ml amphotericin B under a humidified atmosphere of 95% air and 5% CO_2_.

### Establishment of Stable TGF-β1 Transfectants

A cDNA clone encoding full-length mouse TGF-β1 mRNA (GenBank accession no. BC013738) in the pCMV-SPORT6 vector was purchased from OpenBiosystems (Huntsville, AL) and subcloned into pIRES2-AcGFP1 vector (Clontech, Inc. Palo Alto, CA). The IRES2-AcGFP1 vector harboring TGF-β1 was then transfected into SCCVII cells using Lipofectamine 2000 reagent (Life Technologies, Inc. Grand Island, NY). TGF-β1 transfectants were selected by culture for 2 weeks in medium containing 400 μg/ml G418 (Life Technologies, Inc.); the resistant clones were then obtained using the method of limiting dilution. As a negative control, SCCVII cells were transfected with pIRES2-AcGFP1 vector without the inserted TGF-β1 cDNA. The levels of TGF-β1 expression in the stable transfectants were then determined using RT-PCR and an ELISA (R&D Systems Inc., Minneapolis, MN). For RT-PCR, total RNA was isolated from the samples using a Fast RNA Kit Green (Qbiogene, Carlsbad, CA) according to the manufacturer's instructions. After quantifying the isolated RNA using a spectrophotometer, 1-μg aliquots were reverse transcribed using Superscript II reverse transcriptase (Gibco BRL, Gaithersburg, Md.,). The following primer sets were used: for TGF-β1, 5'-ATCTCGAGCTCCGCCATGCCGCCCTCGGGG-3' (forward) and 5'-TCGACTGCAGAATTCTCAGCTGCACTTGCA-3' (reverse); for AcGFP1, 5'-GAGCTGTTCACCGGCATCGT-3' (forward) and 5'-GATGGGGGTATTCTGCTGGT-3' (reverse).

### Cultured bone marrow-derived DCs

Bone marrow-derived DCs (bmDCs) were generated using the method previously described by Labeur et al., with some modification [16]. Briefly, bone marrow was collected from the tibias and femurs of male C3H/He N mice, passed through a 100-μm nylon mesh to remove small pieces of bone and debris, resuspended in CM, and plated in tissue culture dishes for 2 h. Nonadherent cells were collected and plated at a density of 2 × 10^6 ^cells/well in 6-well plates containing 1 ml of CM. Then on days 0, 3 and 5, two-thirds of the medium were replaced with CM containing 20 ng/ml recombinant murine GM-CSF (Peprotech, Rocky Hill, NJ). By day 8 of culture, most of the nonadherent cells had acquired typical DC morphology, and those cells were used as the source of bmDCs. For in vitro experiments, 1 μg of lipopolysaccharide (LPS; Sigma-Aldrich Flanders NJ) was added to the CM on day 7; then after an additional 48 h the mature bmDCs were used. At the end of the procedure, DC purity was assessed based on CD11c expression using single color flow cytometry and was found to be 90% or greater.

### TDLN cell preparation

To prepare TDLNs, tumor cells (1 × 10^6 ^cells/mouse) were inoculated unilaterally into the ears of C3H/He N mice. Fourteen days after inoculation, the mice were killed, and the neck lymph nodes from the side bearing the ear tumor (TDLNs) and from the side without the tumor (non-TDLNs) were aseptically excised. Lymphocyte suspensions were then prepared by teasing apart the nodes to release the cells and then passing the cell suspension through a 100-μm nylon mesh. Erythrocytes were lysed using ACK cell lysis buffer (0.15 M N_4_HCl, 10 mM KHCO_3 _and 0.1 mM EDTA). The cells were then washed and suspended in PBS containing 1% FBS and 2 mM EDTA.

### CFSE labeling of DCs

bmDCs isolated from C3H/He N mice were used as the source of donor DCs in the transfer experiments. Cells were resuspended in PBS at a concentration of 10^7 ^cells/ml and incubated with carboxyfluorescein diacetate succinimidyl ester (CFSE; Molecular Probes Eugene, OR) at a final concentration of 5 μM for 8 min at 37°C, followed by two washes with RPMI 1640 medium containing 10% FCS. Cell division was assessed using flow cytometry by monitoring the dilution of CFSE labeling.

### Injection of bmDCs

Labeled bmDCs were injected into the tumors 13 days after tumor cell inoculation. Each tumor was injected with 1 × 10^6 ^bmDCs in 100 μl of PBS. The TDLNs were then harvested 24 h after injection, and the numbers of bmDCs within the harvested nodes were counted using flow cytometry.

### Flow cytometry

Spleens and TDLNs were excised at the indicated times after tumor cell inoculation. Each sample from an individual mouse was separately prepared and analyzed; i.e., there was no pooling of lymph node cells. Flow cytometric analysis was performed using a Cytomics FC500 (Beckman Coulter, Fullerton, CA). For analysis of DCs, samples were stained with PE-conjugated anti-CD11c and FITC-conjugated anti-CD86 (BD PharMingen, San Diego, CA). In each sample, 100,000 events were routinely acquired and analyzed using a Cytomics FC 500 with CXP Software (Beckman Coulter) to determine the percentage of DCs and CFSE+ bmDCs within the lymph nodes of each clone. Samples from at least ten individual mice were analyzed for each time point unless otherwise stated.

### Quantitative real-time PCR

The primer sequences used to amplify murine TGF-β1 mRNA were 5'-TGGAGCAAC ATGTGGAACTC -3' (left) and 5'-GTCAGCAGCCGGTTACCA -3' (right), and Universal Probe Library #72 (Roche Diagnostics, Mannheim, Germany). All of the amplifications were performed with Light cycler 480 systems (Roche Diagnostics) in a 20-μl final volume, for 45 cycles of denaturation at 95°C for 10 s, annealing at 60°C for 30 s and elongation at 72°C for 1 s. As an internal control, we also amplified murine β-actin mRNA (GenBank accession no. M12481.1) using primers 5'-CTGGCTCCTAGCACCATGA -3' (left) and 5'-ACAGTGAGGCCAAGATGGAG -3' (right) and Universal Probe Library #63 (Roche Diagnostics). After proportional background adjustment, the fit point method was used to determine the cycle in which the log-linear signal was distinguishable from the background, and that cycle number was used as the crossing-point value. Levels of murine TGF-β1 mRNA were then normalized to those of β-actin.

### Analysis of TDLN metastasis

To assess lymph node metastasis, real-time PCR analysis of AcGFP1 mRNA expression was carried out using a Light Cycler 480 (Roche Diagnostics). pIRES2-AcGFP1 vector mRNA was amplified using primers 5'-TGATCTACTTCGGCTTCGTG -3' (left) and 5'-CACTTGTACAGCTCATCCATG C -3' (right) and Universal Probe Library #70 (Roche Diagnostics). In addition, to further confirm the result, metastasis was assessed based on immunohistochemical staining using anti-AcGFP1 (Clontech Laboratories) and goat polyclonal anti-cytokeratin (CK)-19 antibodies (Santa Cruz Biotechnology, Inc, Santa Cruz, CA, USA).

### Statistics

Values are expressed as means ± SD. Groups were compared using one-way ANOVA in combination with Dunnette's methods and paired t test. Values of p < 0.05 were considered significant.

## Results

**Figure 1:**
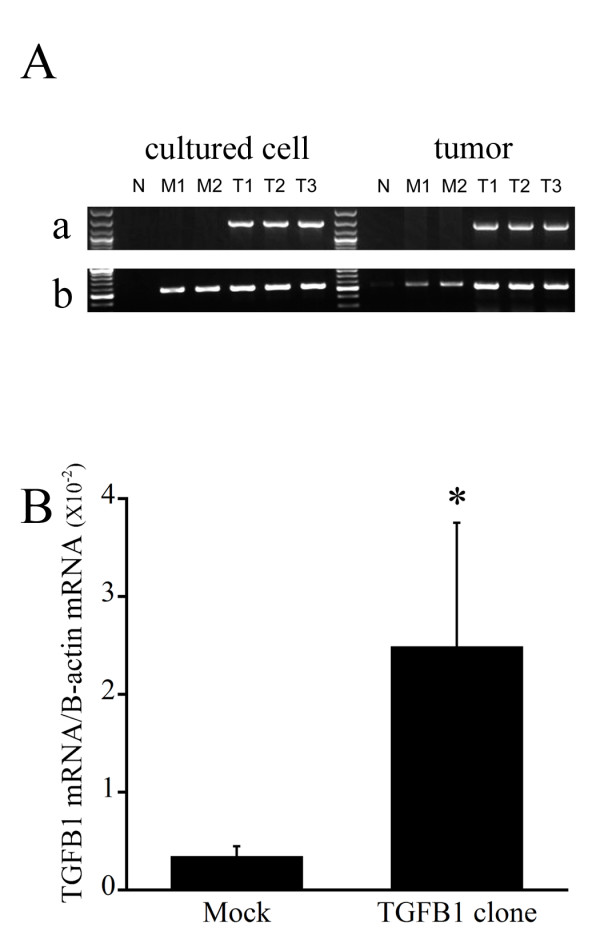
**Characterization of TGF-β1 transfectant clones**. TGF-β1 gene transfection was confirmed by RT-PCR and real-time RT-PCR. A, Expression of TGF-β1 and AcGFP1 mRNA was assessed by RT-PCR. Electrophoresis gels (a and b) show the expression of TGF-β1 and AcGFP1 mRNA, respectively. M1 and M2, mock; T1, T2 and T3, TGF-β1 transfectant clone; N, negative control (SCCVII cells). B, Relative levels of murine TGF-β1 mRNA were determined by semi-quantitative real-time RT-PCR. Levels of TGF-β1 mRNA were normalized to those of β-actin mRNA and were found to be significantly higher in TGF-β1 transfectants.

**Figure 2:**
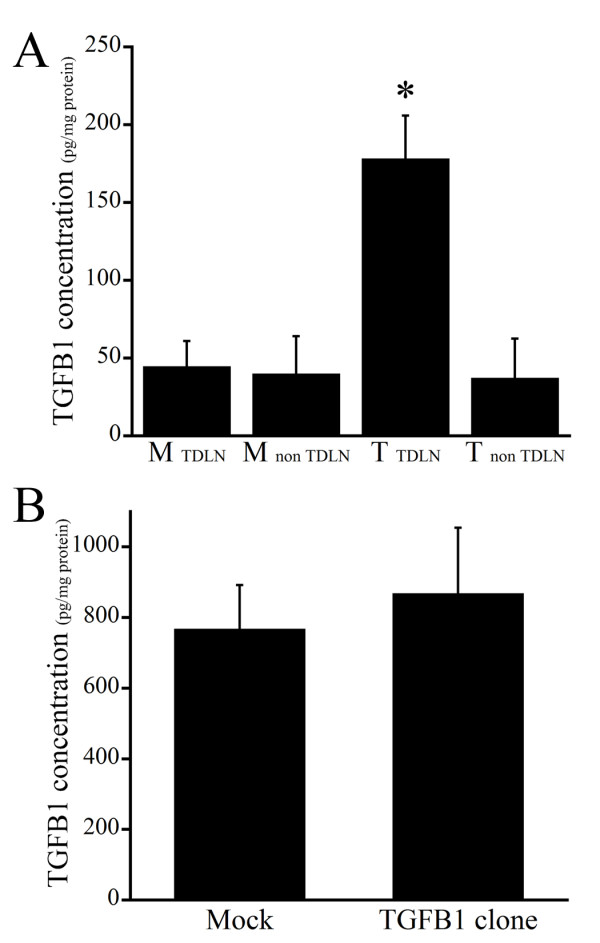
**Concentrations of TGF-β1 in tumor draining lymph nodes**. A, TGF-β1 levels in tumor-draining lymph nodes (TDLNs) and the contralateral nodes (non-TDNLs) in the same mice were assessed using an ELISA. Prior to inoculation, tumor cells were transfected with either TGF-β1 gene or empty vector (mock). Note that in mice inoculated with TGF-β1-transfectants, TGF-β1 levels were higher in TDLNs than non-TDNLs. TGF-β1 levels were also higher in TDLNs draining TGF-β1-expressing tumors than tumors not expressing TGF-β1. B, Serum TGF-β1 levels measured in the same mice as in panel A. Serum TGF-β1 levels did not differ among the groups. *P < 0.05. n = 5 in each group.

After stably transfecting SCCVII cells with murine TGFβ1 cDNA, we initially confirmed the overexpression of TGF-β1 protein by the transfectants. Using RT-PCR with primers for full-length TGF-β1 or AcGFP1 gene, we confirmed the presence of two empty vector-transfected controls (M1, M2) and three TGF-β1-transfected clones (T1, T2, T3) (Figure 1A). When levels of TGF-β1 mRNA were measured using real time PCR (Figure 1B), tumors in mice inoculated with a TGF-β1 transfectant clone showed significantly higher levels of TGF-β1 mRNA than those inoculated with a mock transfectant. In addition, when levels of TGF-β1 protein were measured in cultured cells using ELISAs (Table 1), only TDLN lysates from mice bearing a TGF-β1-expressing tumor showed high levels of TGF-β1 (Figure 2A). By contrast, serum TGF-β1 levels did not differ between mice bearing tumors that expressed TGF-β1 and those did not (Figure 2B).

**Table 1:** Level of TGF-β1 expression in SCCVII cells measured using an ELISA

Cultured cell supernatants	TGF-β1 concentration (pg/mg protein)	Statistics
Wild	183.31 ± 16.91	

Mock transfectants		
1	216.39 ± 6.33	
2	213.94 ± 10.04	

TGF-β1 transfectants		
clone 1	541.35 ± 7.67	P < 0.01
clone 2	392.06 ± 8.65	P < 0.01
clone 3	380.12 ± 20.12	P < 0.01

**Figure 3:**
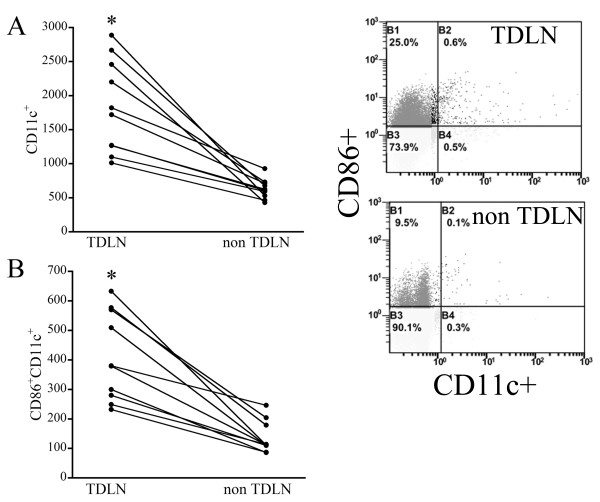
**Increases in the number and biological activity of DCs within TDLNs in wild SCCVII tumor-bearing mice**. A, Numbers of CD11c+ DCs in TDLNs and non-TDLNs on day 14 after tumor inoculation. B, Numbers of CD11c+CD86+ mature DCs in TDLNs and non-TDLNs. The immune response of DCs to tumor antigen was higher in TDLNs than non-TDLNs. *P < 0.05. n = 10 in each group.

To begin assessing DC-mediated immunity in this model, we used flow cytometry to determine the numbers and phenotypes of DCs within the TDLNs and non-TDLNs from wild SCCVII tumor-bearing mice on day 14 after tumor implantation. Figure 3A shows that TDLNs from these mice contained approximately 1.5 to 5 times as many CD11c+ DCs as non-TDLNs. Numbers of CD11c+CD86+ mature DCs were also increased 1.5 to 5 times within TDLNs, as compared to non-TDLNs (Figure 3B). Clearly, the immune response to tumor antigen was higher in TDLNs than in non-TDLNs.

**Figure 4:**
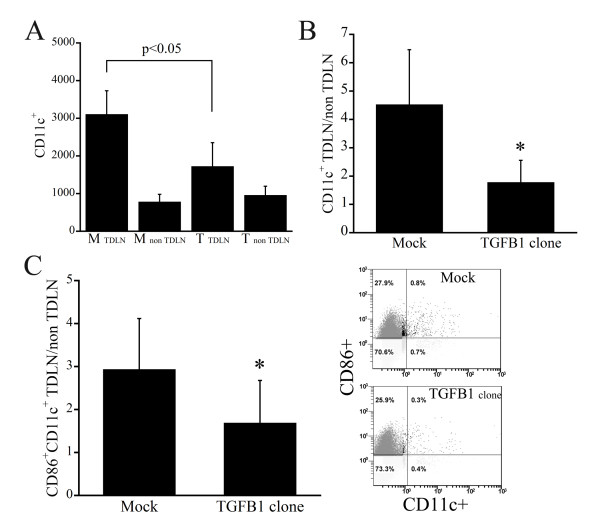
**Tumor-derived TGF-β1 reduces the number of DCs within TDLNs**. A, Numbers of CD11c+ DCs in TDLNs and non-TDNLs from mice inoculated with TGF-β1-tranfected or mock-transfected tumor cells. B, TDLN/non-TDLN ratios for CD11c+ DCs in mice inoculated with TGF-β1-transfected or mock-transfected cells. C, To determine the maturation status of DCs within TDLNs, numbers of CD11c+ and CD86+ DCs were counted, after which the TDLN/non-TDLN ratio for CD11c+CD86+ DCs was calculated. * P < 0.05.

To assess the inhibition of DC migration into TDLNs by tumor-derived TGF-β1, we used flow cytometry to count the numbers of DCs within TDLNs and non-TLDNs. We found that migration of DCs into TDLNs was inhibited in mice inoculated with the three TGF-β1-expressing clones, resulting in a significant reduction in the numbers of CD11c+ DCs within TDLNs (Figure 4A). By contrast, there was no significant difference between the numbers of CD11+ DCs in non-TDLNs from mice inoculated with mock or TGF-β1 transfectants. To identify the maturation status of the DCs within TDLNs, we also counted the numbers of CD11c+ and CD86+ DCs. We found that the TDLN/non-TDLN ratio for both CD11c+ cells and CD86+CD11c+ mature DCs was reduced in mice inoculated with TGF-β1-expressing clones (Figure 4B, C).

**Figure 5:**
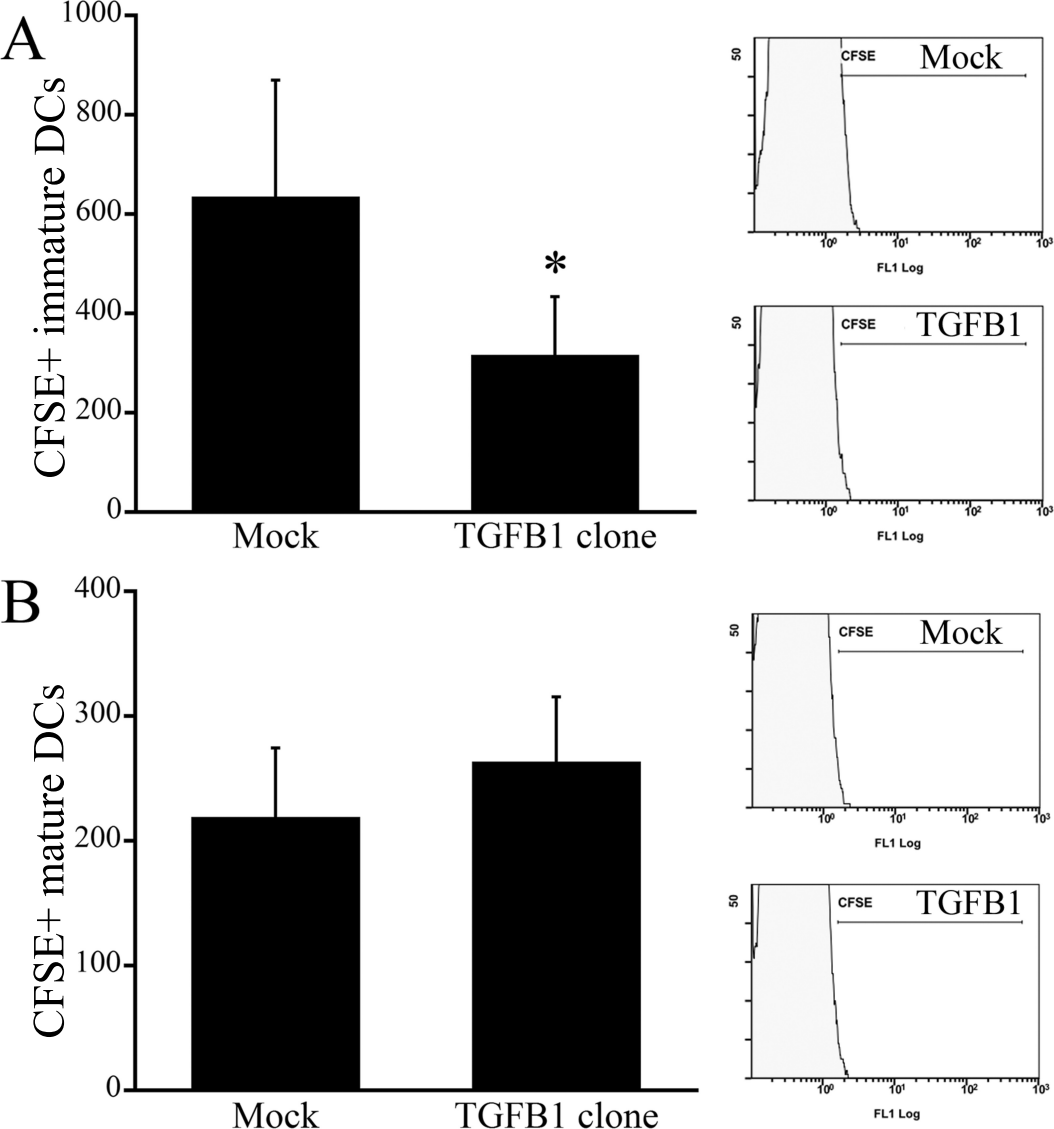
**Tumor-derived TGF-β1 suppresses migration of immature DCs from tumors to TDLNs**. A, To assess migration of DCs from tumors to TDLNs, cultured bone-marrow dendritic cells (bmDCs) were labeled with CFSE and injected into the tumors. Shown are numbers of CFSE-labeled bmDCs within TDLNs counted by flow cytometry 24 h after injection. B, To clarify the maturation status of the migrated bmDCs, untreated immature CFSE-labeled bmDCs and LPS-treated mature CFSE-labeled bmDCs were injected. Note that the numbers of immature bmDCs migrating from TGF-β1-transfected tumors was lower than from mock-transfected tumors, whereas there was no significant difference between the numbers of migrated mature bmDCs. n = 10 in each group. LPS, lipopolysaccharide.

To further clarify the mechanism underlying the reduction in the numbers of DCs within TDLNs, we injected the tumors with CFSE-labeled bmDCs and then counted the numbers of labeled cells within the TDLNs. With this method, we were able to distinguish migrated CFSE-labeled bmDCs from autologous DCs within TDLNs. Flow cytometric analysis of the TDLNs showed that significantly fewer immature (no added LPS) CFSE+ bmDCs migrated from TGF-β1-expressing tumors than from mock-transfected tumors (Figure 5A). By contrast, the total numbers of mature CFSE+ LPS-induced bmDCs did not significantly differ between TDLNs draining mock-and TGF-β1-transfected tumors (Figure 5B). Thus, TGF-β1 suppressed the acquisition by immature DCs of migratory capacity toward lymph nodes.

**Figure 6:**
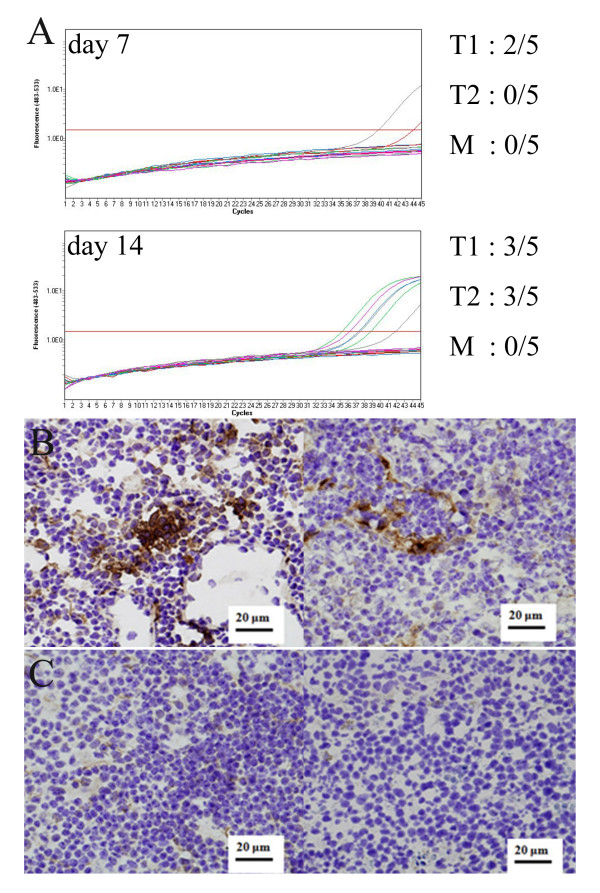
**Tumor derived TGF-β1 induced tumor metastasis in TDLNs**. A, To evaluate tumor metastasis to TDLNs, expression of AcGFP1 mRNA within TDLNs was assessed by RT-PCR. B, Metastasis was confirmed by immunohistochemical detection of CK19 and AcGFP1 within TDLNs draining TGF-β1-expressing tumors (left panel, clone 1; right panel, clone 2). C, Immunohistochemical detection of CK19 and AcGFP1 in TDLNs draining mock-transfected tumors. Note the absence of metastasis in TDLNs draining tumors not expressing TGF-β1.

Finally, to assess TDLN metastasis, we performed real time PCR analysis of AcGFP1 expression in TDLNs draining mock-and TGF-β1-transfected tumors. By day 7 after implantation, metastasis was evident in TDLNs from 2 of 5 mice inoculated with TGF-β1 transfectant clone-1. By day 14, metastasis was detected 3 of 5 TDLNs from mice implanted with TGF-β1 transfectant clone-1 and in the same number of nodes from mice implanted with TGF-β1 transfectant clone-2. On the other hand, no metastasis was detected in TDLNs from mice implanted with mock-transfected clones (Figure 6A).

To confirm the metastasis, we immunohistochemically stained TDLNs with anti-AcGFP1 and anti-CK-19 antibodies. On day 14, AcGFP1+ and CK-19+ cell clusters were found in TDLNs from mice implanted with TGF-β1 transfectant clone-1 or clone-2 (Figure 6B). However, no AcGFP1+ or CK-19+ clusters were detected in TDLNs from mice implanted with a mock-transfectant clone (Figure 6C). Apparently, expression of TGF-β1 by tumor cells increases the likelihood of TDLN metastasis.

## Discussion

In this report we demonstrated that overexpression of TGF-β1 by tumor cells increased the likelihood of metastasis to TDLNs. We also demonstrated that the overexpressed TGF-β1 inhibited DC migration from tumors into TDLNs. Together, these findings suggest that inhibition of DC migration toward TDLNs by tumor-derived TGF-β1 facilitates lymph node metastasis in TDLNs.

Our observation that TGF-β1-expressing tumor cells metastasized to TDLNs is consistent with the clinical evidence, which shows that high levels of TGF-β1 are related to the lymph node metastasis [17,18]. TGF-β plays a critical dual role in the progression of cancer. During the early phase of tumor progression, TGF-β acts as a tumor suppressor. Later, however, TGF-β promotes processes that support tumor progression, including tumor cell invasion, dissemination and immune evasion [19]. In this study we also demonstrated that overexpressed TGF-β1 inhibits DC migration from tumors to TDLNs. Because DCs play a key role in cell-mediated immunity by acting as an antigen-presenting cell, a TGF-β1-induced reduction in DC migration into TDLNs would be expected have an immunosuppressive effect within TDLNs, thereby promoting tumor metastasis into TDLNs.

Following injection of CFSE-labeled DCs into SCCVII tumors, the numbers of labeled DCs that migrated into TDLNs from tumors expressing TGF-β1 was lower than the numbers that migrated from tumors not expressing TGF-β1. TGFβ1 can immobilize DCs, interfering with their migration and thus the transport of antigen to draining lymph nodes for presentation to adaptive immune cells. Although we do not provide direct evidence of the mechanism by which TGF-β1 inhibits DC migration toward TDLNs in this study, Weber et al. reported that TGFβ1 inhibits DC migration from skin tumors to draining lymph nodes, based on the disappearance of E-cadherin+ DCs from draining lymph nodes consistent with our results [20]. Moreover, Ogata et al. demonstrated that TGF-β1 not only inhibits expression of CCR7 on DCs, it also inhibits chemokine-mediated DC migration in vitro [17]. We therefore conclude that tumor-derived TGF-β1 inhibits DC migration from tumors to TDLNs.

In further investigating the role of TGF-β in metastasis, mice models of metastasis have revealed that systemic inhibition of the TGF-β signaling pathway negatively affects metastasis formation. Consistent with our hypothesis, several independent groups by Padua D et al. and reference therein [21] have found that small-molecule inhibitor of the TGF-β receptors (TGFBR) type I with a human breast cancer cell line, and TGF-β antagonist of the soluble TGFBR2 in a transgenic model decrease the cancer's metastatic capacity. These results illustrate the capacity to target the TGF-β pathway in order to effectively inhibit metastatic events [21]. However, given the clinical and experimental evidence that TGF-β acts as a tumor suppressor, other groups have argued that TGF-β functions as an inhibitor of epithelial tumor growth and metastasis. In the example, loss of TGFBR2 in mammary epithelial cells or fibroblasts increased tumor formation and enhanced many markers of tumor progression [22]. TGFBR2 knockout animals developed significantly more pulmonary metastases [23]. Interestingly, TGFBR2 knockout tumors have high levels of TGF-β1 most likely secreted by myeloid suppressor cells [24]. These authors argue that the TGF-β1 may provide an additional boost to tumor progression by dampening the immune response to the tumors. Here we provide new direct evidence for such an effect.

In the present study we did not directly prove that the reduction in DCs migration causes tumor metastasis into TDLNs. In addition to its immunosuppressive effect, TGF-β1 upregulates cell motility and invasiveness, as well as epithelial-to-mesenchymal transition [19]. These effects may have also promoted lymph node metastasis in our study. Further investigation will be needed to more precisely define the role of tumor-derived TGF-β1 in tumor lymph node metastasis.

## Conclusions

In sum, we have shown that overexpression of TGF-β1 by tumor cells promotes tumor metastasis into TDLNs, most likely by inhibiting DC migration from tumors towards TDLNs. This immunosuppressive effect would be expected to promote lymph node metastasis in patients with malignant disease.

## Competing interests

The authors declare that they have no competing interests.

## Authors' contributions

KI did animal experiments, flow cytometry work, and analyzed data and wrote the paper. YM designed the research. SK and TS made TGF-β1 transfected SCCVII cell line. MI, HS, YS and SM performed contributed data analysis. JO contributed experimental design and data analysis. All authors read and approved the final manuscript.
